# Feasibility and Safety of Targeting Mitochondria Function and Metabolism in Acute Myeloid Leukemia

**DOI:** 10.1007/s40495-024-00378-8

**Published:** 2024-10-04

**Authors:** Patryk Firmanty, Monika Chomczyk, Shubhankar Dash, Marina Konopleva, Natalia Baran

**Affiliations:** 1 Department of Experimental Hematology, Institute of Hematology and Transfusion Medicine, Warsaw, Poland; 2 Department of Oncology, Albert Einstein College of Medicine, Bronx, NY, USA; 3 Department of Leukemia, The University of Texas, MD Anderson Cancer Center, Houston, TX, USA

**Keywords:** Acute myeloid leukemia, Mitochondrial metabolism, Metabolic reprogramming, Safety, Therapeutic interventions

## Abstract

**Purpose of Review:**

Acute myeloid leukemia (AML) is a clonal blood neoplasm with dismal prognosis. Despite the introduction of many novel targeted agents, cytotoxic chemotherapy has remained the standard of care for AML. Differences in mitochondrial metabolism between normal and leukemic cells can be targeted by novel AML therapies, but these agents require a comprehensive efficacy and cytotoxicity evaluation.

**Recent Findings:**

Metabolic alterations in AML blasts increase their sensitivity to therapies targeting mitochondrial metabolism. Targeting altered mitochondrial metabolism, that is crucial for leukemia cell growth and survival, could be a breakthrough in AML treatment. Therefore, BH3 family proteins, mitochondrial complexes, the tricarboxylic acid cycle, and amino acid (AA) and fatty acid metabolism are common treatment targets in AML. Although many drugs targeting these vulnerabilities showed acceptable safety profiles and promising efficacy in preclinical studies, clinical trials often do not confirm these results limited by narrow therapeutic window. The most effective regimens are based on drug combinations with synergistic or additive activity.

**Summary:**

In this review, we present an overview of the most recent studies targeting mitochondrial metabolism in AML. We highlight that targeting of the specific energy metabolism dependencies of AML blasts provides an opportunity to achieve long-term responses with a reasonable safety profile. We emphasize that currently used drugs and their combinations display dose-limiting toxicities or are not efficient enough to completely eradicate leukemic stem cells. Thus, further studies of complex metabolic rewiring of leukemia cells before and after combinatorial therapies are warranted.

## Introduction

Acute myeloid leukemia (AML) involves a rapid clonal proliferation of immature myeloid progenitor cells (blasts) in the bone marrow and peripheral blood [1]. Expansion of leukemic blasts subsequently compromises healthy hematopoiesis and leads frequently to coagulopathy, pancytopenia, immunosuppression, and ultimately increased risk of infections and death [1, 2].

Despite the introduction of many novel targeted agents, cytotoxic chemotherapy has remained the standard of care for younger adults with AML. The 5-year overall survival rates for adult patients are low due to advanced age, comorbidities, and frequently occurring inherent or acquired chemoresistance [3]. Therefore, an urgent need for new, safe, well-tolerated, and target-specific therapies persists. Over the past three decades, a search for specific targets addressing leukemia and extensive work on genetic and transcriptomic characterization of leukemia biology have been undertaken. This work has revealed genetic alterations and other characteristics associated with specific subtypes of AML, along with subtype-specific outcomes, and led to the development of several preclinical and clinical trials of drug candidates to target these alterations [4].

Nevertheless, standard of care treatments, alone or even combined with novel compounds, still fail to target leukemic stem cells (LSCs), which are responsible for disease persistence, recurrence, and poor clinical outcomes [3, 5]. LSCs display unique transcriptional and epigenetic signatures that make the LSCs distinct not only from bulk AML cells but even more from their normal hematopoietic counterparts [6-8]. LSCs also diverge from hematopoietic stem cells (HSCs) by their reprogrammed metabolism’s distinct metabolic signature, which presents an attractive vulnerability for new selective therapies. In this review, therefore, we describe general differences in mitochondrial metabolism between normal and leukemic cells and present these differences as a potential target in the context of current and future AML drug development. Finally, we discuss perspectives and challenges associated with these strategies.

## Mitochondrial Metabolism in Healthy Cells and Malignant AML

### Energy Metabolism

In the cellular metabolism of leukemia cells, several alterations occur to meet the bioenergetic and biosynthetic needs, supporting both energy production and biomolecule synthesis. These metabolic shifts are mainly triggered by mechanisms that instigate signaling pathways and modulate the expression of diverse genes related to metabolism control.

In normal HSCs, the main source of energy generation comes from glycolysis, whereas LSCs are metabolically dependent on oxidative phosphorylation (OXPHOS) and electron transport chain (ETC) activity [9, 10]. In glycolysis, glucose in cytoplasm is converted to pyruvate, and further to acetyl-CoA, which enters mitochondrial tricarboxylic acid cycle (TCA), resulting in the generation of NADH and FADH2 [9, 11]. Despite the presence of oxygen, pyruvate can be converted into lactate, which frequently occurs in a subset of chemotherapy-resistant AML cells upregulating a glycolysis or following OXPHOS-inhibiting therapy [9, 10, 12].

Acetyl-CoA can be also derived from the mitochondrial aerobic process of fatty acid degradation known as fattyacid oxidation (FAO). Since FAO metabolism facilitates asymmetric cell division of HSCs, HSCs maintain FAO metabolism at low levels to preserve dormancy [11, 13]. Conversely, in LSCs an enrichment in FAO-related genes has been associated with increased resistance to therapy with BCL-2 inhibitor venetoclax [9, 14].

**Fig. 1:**
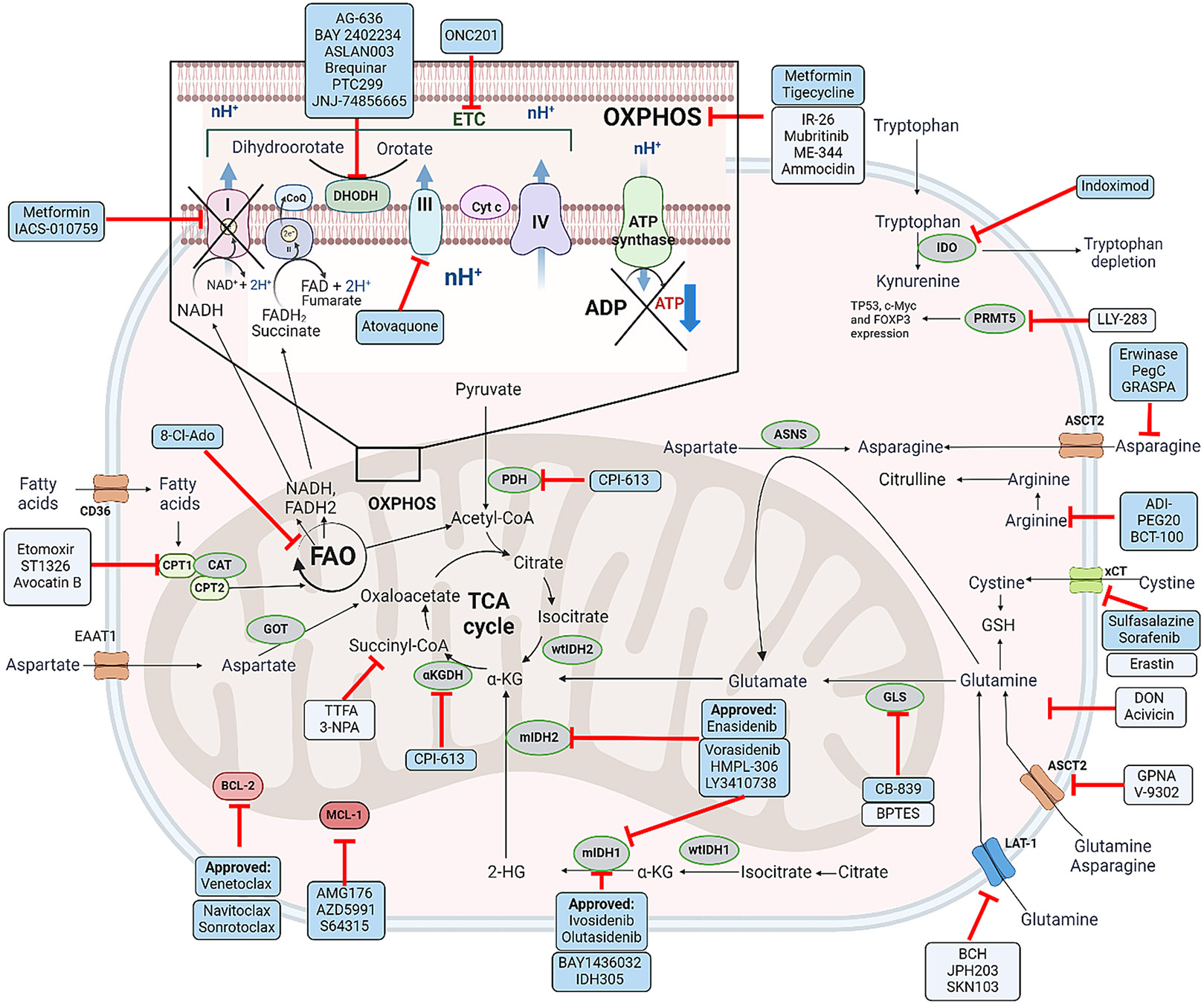
Preclinically and clinically tested compounds targeting mitochondrial metabolism in AML. Compounds targeting the intracellular or extracellular activity of enzymes or transporters involved in amino acids metabolism taking part in mitochondria are shown. The specific function of each target is shown. Light blue boxes demonstrate compounds investigated in preclinical studies. Dark blue boxes show drugs which have already entered clinical trials. Drugs described as “approved” have been approved by the FDA to use in AML treatment. 8-Cl-Ado: 8-chloroadenosine; ADI-PEG20: arginine deiminase PEGylated 20; αKG: alpha-ketoglutarate; αKGDH: alpha-ketoglutarate dehydrogenase; ASCT2: alanine, serine, cysteine transporter 2; BCL-2: B-cell lymphoma 2; BPTES: Bis-2-(5-phenylacetamido-1,2,4-thiadiazol-2-yl)ethyl sulfide; CD36: cluster of differentiation 36; CPT1: carnitine palmitoyltransferase 1; DHODH: dihydroorotate dehydrogenase; DON: 6-diazo-5-oxo-L-norleucine; FAO: fatty acid oxidation; FADH2: flavin adenine dinucleotide; GDH: glutamate dehydrogenase; GLS: glutaminase; GLUT1: glucose transporter type 1; GSH: glutathione; IDH1/2: isocitrate dehydrogenase 1 and 2; IDO: indoleamine 2,3-dioxygenase; LAT-1: large neutral amino acid transporter 1; MCL-1: myeloid cell leukemia 1; NADH: nicotinamide adenine dinucleotide; OXPHOS: oxidative phosphorylation; PDH: pyruvate dehydrogenase; PRMT5: protein arginine methyltransferase 5; ROS: reactive oxygen species; TCA cycle: tricarboxylic acid cycle; xCT: cystine/glutamate transporter

In leukemic cells, glutamine becomes essential for both energy generation and biosynthesis source [15-17]. It is converted by glutaminases to glutamic acid and then to α-ketoglutarate, which enters the TCA cycle, contributing to net production of energy molecules needed in the ETC (Fig. 1) [15, 18]. LSCs’ survival depends on the metabolism of glutamine and other amino acids to sustain OXPHOS; therefore, depleting AAs in culture media leads to apoptosis of LSCs, sparing the majority of normal HSCs [18, 19].

OXPHOS is a process of ATP generation in which electrons from NADH and FADH2 are transferred to oxygen across five mitochondria membrane ETC complexes [12, 20]. Several components of ETC complexes I and V are more abundant in LSCs than in HSC [20-23]. HSCs tend to be glycolytic in the dormant state and switch to a more oxidative phenotype upon the process of cell differentiation [24, 25]. LSCs, in contrast to bulk AML cells, are unable to drive glycolysis and mainly rely on OXPHOS [10, 11]; even upon OXPHOS inhibition they cannot switch back to glycolysis for energy demand [12, 18, 20, 26, 27]. In summary, in contrast to HSCs, LSCs rely on OXPHOS and depend on AA and FA metabolism to maintain high OXPHOS levels.

### Interactions between Mitochondrial Metabolism, Biogenesis and Mitophagy in AML

Mitochondrial homeostasis is preserved by the coordination between two processes: mitochondrial biogenesis and mitophagy [28, 29]. Mitochondrial biogenesis can be defined as the growth of preexisting mitochondria and the increase of mitochondrial number, tightly regulated by a tissue-specific transcriptional coactivator, peroxisome proliferator-activated receptor gamma coactivator 1-alpha (PGC-1α) [29]. Using PGC-1α, invasive cancers enhance mitochondrial biogenesis resulting in increased OXPHOS [29, 30], while silencing of PGC-1α decreases tumor growth and its invasive potential [30]. These findings therefore identify PGC-1α as a potential therapeutic target of OXPHOS-dependent cancers [30]. Mitophagy regulates mitochondrial quality and quantity control and is attributed to the PTEN-induced putative kinase (PINK1)-Parkin pathway and mitochondrial protein ubiquitination [28]. However, recent reports highlight the importance of receptor-mediated mitophagy in a Parkin-independent manner, which is mediated by mitochondrial proteins such as BCL-2interacting protein 3 (BNIP3) that directly interact with microtubule-associated proteins 1 A/1B light chain 3 (LC3) with no need for ubiquitination. PGC-1α is also increased in bone marrow mesenchymal stem cells (MSCs) followed culture with leukemic cells, indicating PGC-1α activation as a requirement for pro-tumoral mitochondrial transfer from MSCs to leukemic cells [31].

Interestingly, PGC-1α can activate the PINK1-Parkin pathway indirectly via the estrogen-related receptor alpha (ERRα)-sirtuin 3 (SIRT3) pathway, while its downregulation can upregulate BNIP3, inducing a PINK1-Parkin-independent mitophagy [32], indicative of PGC-1α’s essential role in mitophagy regulation [32].

**Fig. 2:**
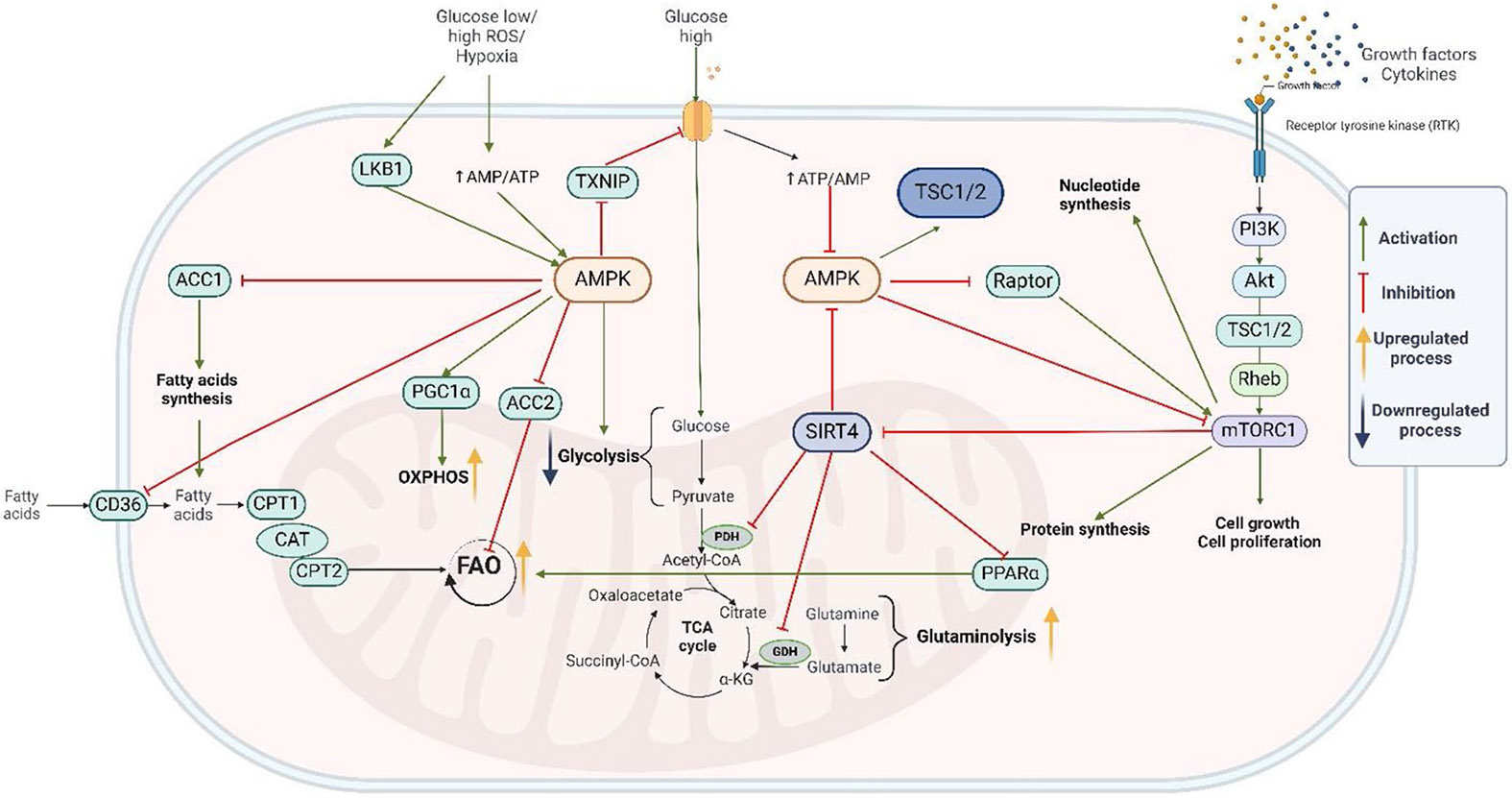
Metabolic changes and metabolic related signaling in AML. This figure encapsulates the complex interplay of metabolic pathways and signaling networks that contribute to the metabolic reprogramming observed in AML cells. Yellow arrows represent metabolic processes upregulated and downregulated in AML blasts. Cellular metabolism is mainly regulated by AMPK and mTOR. AMPK is activated by increased AMP/ATP ratio or LKB1 (activated by low glucose level, high ROS level, or hypoxia) and lead to upregulation of cellular catabolism. AMPK upregulates glucose uptake, glycolysis, the TCA cycle, glutaminolysis, OXPHOS, and FAO. AMPK also downregulates anabolic processes such as fatty acid, protein, and nucleotide synthesis. AMPK upregulates glucose uptake by GLUT1 through TXNIP inhibition that inhibits GLUT1. AMPK also upregulates FAO through ACC2 inhibition that inhibits FAO. AMPK also indirectly upregulates FAO through SIRT4 inhibition. (SIRT4 inhibits PPARα that upregulates FAO) AMPK also upregulates OXPHOS through PGC1α activation. Interestingly, AMPK might control fatty acid uptake through CD36 inhibition. AMPK inhibits mTOR, which is involved in cellular anabolic processes (AMPK inhibit mTOR-activating Raptor). mTOR can be activated by PI3K activation and further by AKT activation. PI3K can be activated by RTK activation through cytokines and growth factors. Interestingly, SIRT4, which inhibits PDH and GDH (both essential for the TCA cycle), is downregulated by both AMPK and mTOR activity. ACC2: acetyl-CoA carboxylase 2; AMP: adenosine monophosphate; AMPK: AMP-activated protein kinase; ATP: adenosine triphosphate; FAO: fatty acid oxidation; GDH: glutamate dehydrogenase; GLUT1: glucose transporter 1; LKB1: liver kinase B1; mTOR: mechanistic target of rapamycin; OXPHOS: oxidative phosphorylation; TCA: tricarboxylic acid cycle; PDH: pyruvate dehydrogenase; PGC1α: peroxisome proliferator-activated receptor gamma coactivator 1-alpha; PI3K: phosphoinositide 3-kinase; PPARα: peroxisome proliferator-activated receptor alpha; ROS: reactive oxygen species; SIRT4: sirtuin 4; TXNIP: thioredoxin interacting protein

PGC-1α is tidily connected with the activity of adenosine monophosphate-activated protein kinase (AMPK) (Fig. 2) [33]. AMPK activates PGC-1α through phosphorylation, resulting in increased production of OXPHOS subunits, TCA cycle enzymes, and FAO enzymes, which activate the mitochondrial biogenesis pathways [33]. Notably, AMPK influences both mitophagy and mitochondrial biogenesis [33].

AMPK affects many metabolic pathway enzymes that mediate mitochondrial metabolism and has demonstrated both tumor suppressing, and tumor promoting activity [34]. Notably, knockout of AMPK in HSCs had only a limited impact on HSC function [35], while LSCs require AMPK for survival by maintaining low reactive oxygen species (ROS) levels [36].

Another signaling pathway integrating signals related to energy status and nutrient availability involved in the coordination of mitophagy and mitochondrial biogenesis is mammalian target of rapamycin complex 1 (mTORC1) [8, 37]. mTORC1 signaling pathway is activated upon nutrient abundance but undergoes inhibition when nutrient availability is limited, promoting mitochondrial degradation through mitophagy, which is necessary for AML cell survival [37]. Mitophagy finally is an important process for LSCs as it supports the maintenance of low levels of ROS, necessary for self-renewal of AML LSCs [38].

Knockdown of the gene encoding BNIP3L/Nix sensitized leukemic cells to mitochondria-targeted therapies [39]. Also, knockdown of mitochondrial fission protein 1 (FIS1), mitofusins 1 and 2 (MFN1, MFN2), and optic atrophy 1 (OPA1)—which are involved in mitochondrial dynamics—decreased the stem potential of LSCs and disrupted mitochondrial respiration while leaving normal HSCs unaffected [38, 40]. In vitro studies also revealed that targeting dynamic-related protein 1 (DRP1) sensitizes AML cells to venetoclax-induced programmed cell death [41]. Additionally, mitophagy promotes AML cells survival in hypoxic conditions—it stimulated damaged mitochondria elimination and induced transfer of mitochondria derived from mesenchymal stem cells (MSCs) into leukemic cells, contributing to AML cell survival [42]. Given the fact, that mitochondrial biogenesis is required for increased OXPHOS (necessary for LSCs) and the mitophagy and mitochondrial dynamics are essential for maintaining stem potential of LSCs, targeting these processes could be a promising therapeutic strategy for AML.

## Targeting Mitochondrial Metabolism in AML

### BCL2/BH3 Family

One of the key requirements for maintenance of AML cells is the resistance to apoptosis [8, 39, 43-45]. This is achieved by the overexpression of pro-survival B-cell lymphoma 2 (BCL-2) family proteins, which share BCL-2-like homology domains 1–4 (BH1-BH4) [44, 45]. The pro apoptotic proteins of BCL-2 family such as BID, BIM, BAD, NOXA, and PUMA trigger apoptosis by intercalating into the outer mitochondrial membrane via BH3 regions, increasing its permeability and thereby translating cellular stresses into apoptosis initiation [39, 44-46]. The latter is often activated via BCL-2-family-member (such as BAX, BOK, BAK or BCL-XS)-mediated mitochondrial permeabilization induced by standard cytotoxic chemotherapy, providing the rationale for targeting the antiapoptotic members of the BCL-2 family with small molecule drugs [43]. The best studied drugs of the BCL-2 family members inhibitors are venetoclax (ABT-199), and navitoclax (ABT-263) [47-50]. Navitoclax is the first-in-class oral BCL-2/-XL dual inhibitor with antileukemic activity; however, its development has been discontinued due to dose-limiting severe thrombocytopenia [50]. Venetoclax was the first clinically effective BCL-2-targeting drug approved by the US Food and Drug Administration (FDA) for leukemia treatment [47, 51-55]. Venetoclax showed manageable adverse effects (AEs) such as hypokalemia, neutropenic fever, and lymphopenia, with the latter mostly due to heightened BCL-2 priming and sequestration of proapoptotic proteins Bim and Bax in mature lymphocytes but not in other healthy cells [56-60]. Venetoclax demonstrated potency to inhibit mitochondrial respiration and AA metabolism [49, 61, 62]. Interestingly, due to AML cells’ dependency on glutaminolysis, its inhibition activates mitochondrial apoptosis and sensitizes AML cells to venetoclax without impacting HSCs’ function [63, 64]. These metabolic effects of venetoclax were dependent on the integrated stress response and activity of ATF4 transcription factor but independent of BCL-2 inhibition [61]. However, cells that developed venetoclax resistance showed increased OXPHOS activity than before treatment [8, 65], and this activity is targetable by pharmacologic inhibition of mitochondrial protein synthesis with ribosome-targeting antibiotic tedizolid and doxycycline [66]. However, some venetoclax-resistant AML cells use AAs or fatty acids by stimulation of nicotinamide metabolism, displaying a phenotype more resistant to venetoclax than cells only using OXPHOS for energy generation [26].

The modest antileukemic activity of venetoclax and often-observed resistance prompted further studies of its combinations with other active agents such as the hypomethylating agent azacytidine [51]. In the long-term phase 3 VIALE-A study (NCT02993523) in newly diagnosed (ND) AML patients ineligible for intensive chemotherapy (IC), venetoclax and azacytidine significantly prolonged survival compared to azacytidine monotherapy [51]. The composite complete remission (CRc) rate was 66.4%, and the most frequent grade ≥ 3 AEs were thrombocytopenia and febrile neutropenia [51]. Long-term efficacy and safety studies confirmed venetoclax-azacytidine as an improvement in the standard of care for patients with AML who are not eligible for IC. In a similar group of patients ineligible for IC, venetoclax was also administered with low-dose cytarabine (LDAC) [67]. The long-term VIALE-C phase 3 study (NCT03069352) assessed the efficacy and safety of venetoclax and LDAC reported a 48.3%, CRc rate, with grade ≥ 3 AEs including neutropenia, thrombocytopenia, and febrile neutropenia; therefore, the authors recommended this regimen as an effective and safe induction therapy option [67]. Venetoclax was also tested in combination with standard AML induction/consolidation therapy [5]. A phase 2 clinical trial (NCT02115295) in 50 patients with AML reported a CRc of 94% with manageable grade ≥ 3 AEs including febrile neutropenia and ALT elevations [5].

The combination of venetoclax and a small molecule FLT3-tyrosine kinase inhibitor (TKI), quizartinib or gilteritinib, was tested in patients with FLT3 mutations and demonstrated superior efficacy than venetoclax monotherapy [68, 69]. In a phase 2 trial (NCT03661307), the triple combination of venetoclax, quizartinib, and decitabine in patients with FLT3-ITD-mutated AML showed high activity with a 68% CRc rate [69]. The most common non-hematologic grade ≥ 3 AEs were pneumonia and neutropenic fever [69]. Venetoclax was also tested with gilteritinib in relapsed/refractory FLT3-mutated AML in a multicenter clinical trial (NCT03625505), leading to a 75% CRc rate with most common grade ≥ 3 AEs involving febrile neutropenia and decreased white blood cell count [68]. CRc rates and the most common grade ≥ 3 AEs reported in venetoclax-based trials and others showing clinical benefits are summarized in Table 1.

However, in response to BCL-2 inhibition with venetoclax, myeloid malignancies tend to upregulate and depend on other members of BCL-2 family, such as myeloid cell leukemia-1 (MCL-1), as mechanisms of venetoclax resistance [43, 57]. This finding led to the development of selective MCL-1 inhibitors [43, 57, 70]. Further, dual inhibition of BCL-2 and MCL-1 synergistically enhanced apoptosis in AML cells, promoting initiation of clinical trials with drug candidates such as AMG176, AZD5991, or S64315 [43, 57, 70-72]. Mechanistically, MCL-1 inhibitors deregulate OXPHOS and induce mitophagy, suggesting its role is more complex than solely cell death inhibition [73, 74]. MCL-1 inhibitors were well tolerated in some preclinical models; however, cardiac-specific ablation of MCL-1 resulted in decreased functionality of cardiomyocytes, followed by ultrastructural mitochondrial abnormalities and mitochondrial respiratory defects [75]. Due to reported cardiotoxicity and suppression of hematopoiesis, none of Mcl-1 inhibitors have been approved for clinical use and most discontinued development [70, 72]. Besides MCL-1 upregulation, other mechanisms of venetoclax resistance have been reported, indicating a genetically diverse landscape among AML patients and heterogeneity of mechanisms used by cells to overcome venetoclax treatment. Of the latter, four different expression programs and thus clusters of patients were identified [8]. Patients in cluster 1, which is characterized by high PI3K-mTOR signaling, OXPHOS, and fatty acid metabolism, were sensitive to panobinostat, which suppresses OXPHOS and mTORC1 signaling [8]. Cluster 2-like samples show a high proliferation rate along with high rates of NRAS mutations and suppression of HOX genes, while cluster 3 is characterized by high enrichment in TP53 mutations and elevated JAK/STAT pathway activity; these cells were responsive to treatment with ruxolitinib [8]. These results show clonal and phenotypic heterogeneity in venetoclax-resistant AMLs, and genetic/pharmacological testing to select effective combination therapies should be considered [8]. Finally, the next-generation BCL2 inhibitor sonrotoclax (BGB-11417) demonstrated a potent antitumor activity on hematologic cancer cells that are resistant to venetoclax treatment [76]. Sonrotoclax as a monotherapy or in combination with other anticancer agents is under clinical investigation in AML and other hematological malignances (NCT04771130, NCT04277637, NCT05479994, NCT05471843).

### Mitochondrial Complexes

OXPHOS is a major metabolic pathway in AML, and upregulated expression of OXPHOS-related genes is associated with poor prognosis in AML [8, 12, 65, 77, 78]. Elevated OXPHOS activity is a hallmark of cytarabineresistant AML cells [78]. Previous reports suggest a clonal pressure on initially OXPHOS-heterogenous AML towards homogenous high-OXPHOS disease following cytarabine therapy [78]. High-OXPHOS, therefore, could be a promising therapeutic target in primary and refractory/post-treatment AML. Toward this end, clinically applicable OXPHOS inhibitors have been developed, and drugs with known OXPHOS-inhibiting activity have been repurposed [12, 21, 65, 77]. Well-known drugs studied for their specific OXPHOS-inhibitory activity in myeloid leukemia include metformin or phenformin, tigecycline, tamoxifen, atovaquone, IM156, ME-344, IACS-010759, and ammocidin [77, 79-81].

Metformin, a biguanide used to treat diabetes, reduces the risk of certain types of cancer by directly decreasing OXPHOS activity [79, 82]. Its OXPHOS inhibitory activity was tested extensively in clinical trials for solid tumors (NCT03071705, NCT01579812, NCT02496741) but less in AML (NCT01849276), and only modest clinical benefits has been observed [82].

Another biguanide, IM156, has OXPHOS-inhibiting properties [77] but showed limited single-agent activity in a phase 1 trial (NCT03272256), and further clinical trials are focused on combinations with other anticancer agents (NCT05497778) [77].

A preclinical AML xenotransplant study using tigecycline, an antimicrobial that inhibits mitochondrial protein translation, revealed selective reduction of AML tumor burden with no effect on HSC [80]. However, despite its satisfactory safety profile, a phase 1 trial of tigecycline in AML patients fell short because of limited efficacy [81].

Tamoxifen, an estrogen modulator approved for treatment of estrogen receptor-positive breast cancer, showed activity to downregulate the oncogenic JAK-STAT pathway and inhibit mitochondrial complex I, leading to significant clinical benefits in some patients with myeloproliferative neoplasms in a phase 2 trial [83]. Additionally, tamoxifen combined with C6-ceramide induced cell death in an mitophagy-dependent manner in AML cell lines [84]. These results suggest that tamoxifen requires further investigation in AML.

Atovaquone, well-tolerated antimalarial drug, also suppress OXPHOS through mitochondrial complex III inhibition [85, 86]. The favorable side effect profile and strong reduction of AML burden in patient-derived xenograft (PDX) mouse models provide a strong rationale for the incorporation of atovaquone into AML therapy [85, 86]. A clinical trial in pediatric AML patients showed that combining atovaquone with standard chemotherapy was safe and demonstrated OXPHOS suppression [85, 86]. However, when standard dosing is followed, sufficient plasma concentration of atovaquone is often not achieved [85, 86].

Ammocidin, a natural glycomacrolide product that inhibits mitochondrial complex V (ATP synthase) also demonstrated an antileukemic activity in vivo at doses tolerated with minimal toxicity. Ammocidin further synergized with venetoclax in preclinical AML models [87], providing a strong rationale for its testing in clinical trials [88].

Mitochondrial complexes can also be inhibited by newly synthesized agents such as the dye IR-26, which interacts with complex II and V proteins, impaired OXPHOS, and reduced leukemia burden in mouse models without harming vital organs [89]. Besides its antileukemic properties, IR-26 offers a promising strategy for real-time monitoring of treatment efficacy [89].

Mubritinib, a mitochondrial complex I inhibitor, also exhibited potent antileukemic activity in mouse models without impairing normal hematopoiesis [90]. The safety of mubritinib was confirmed in a phase 1 trial in solid tumors (NCT00034281), warranting further investigation in clinical trials as an anticancer agent.

The isoflavone ME-344 inhibits OXPHOS activity and suppresses purine biosynthesis. ME-344 demonstrated antileukemic activity as monotherapy and combined with venetoclax in AML PDX models while sparing normal HSCs [65]. Numerous clinical trials have evaluated ME-344’s safety and antitumor activity in solid tumors, alone and in different combinations (NCT01544322, NCT02100007). ME-344 was well tolerated, but its antitumor efficacy varied across tumor types, limiting indications for further investigation [65].

IACS-010759, a selective small-molecule complex I inhibitor, appeared promising. In AML PDX mouse models, IACS-010759 significantly reduced leukemia burden as monotherapy and combined with standard chemotherapy or venetoclax [15, 91, 92]. This effect was achieved by eradication of chemotherapy-resistant, OXPHOS-dependent LSCs [91]. However, in a phase 1 trial in patients with relapsed or refractory (R/R) AML (NCT02882321), dose-limiting toxicities, including elevated blood lactate and neurotoxicity, were encountered, and clinical trials were halted [12].

Despite high expression of OXPHOS-related genes in AML cells, OXPHOS is also required for intracellular oxygenation and normal physiological processes in healthy cells. Many studies revealed that heart, muscle, kidney, adrenal gland, liver, and brain tissues also have high average expression of OXPHOS genes [12, 93]. Therefore, myalgia, brain toxicity, hepatotoxicity, and cardiotoxicity are common side effects of all OXPHOS inhibitors and further efforts are needed to identify safe antitumor OXPHOS inhibitors [12, 93].

### TCA Cycle

One of the key enzymes of TCA is isocitrate dehydrogenase (IDH), which converts isocitrate to α-ketoglutarate [52, 94-96]. Among three different isoforms, IDH1, IDH2 and IDH3, mutations in IDH1 or IDH2 occur in 20–30% of AML patients resulting in production of neomorphic enzymes, catalyzing abnormal DNA histone methylation and promoting leukemogenesis [96, 97]. Thus, abrogated IDH activity is a target for treatment of R/R IDH-mutated AML, with three FDA-approved IDH inhibitors: ivosidenib (AG-120, IDH1 inhibitor), olutasidenib (FT-2102, IDH1 inhibitor), and enasidenib (AG-221, IDH2 inhibitor) [52, 94-102]. In a clinical trial (NCT02074839), ivosidenib monotherapy demonstrated a 30.4% CRc rate and low frequency of grade ≥ 3 AEs, although some patients appeared treatment-resistant [103]. In preclinical trials, IDH inhibitors showed more potent antileukemic effects in combination with either hypomethylating agents, standard induction chemotherapy, or venetoclax, providing a rationale for clinical trials of these combinatory treatments [52, 94-102]. In a phase 1 trial (NCT02632708), ivosidenib combined with IC demonstrated a 78% CRc rate in patients with ND IDH1-mutant AML [104]. A low frequency of grade ≥ 3 AEs was reported, including hypophosphatemia and prolonged QT interval [104]. These results supported further investigation in a phase 3 trial (NCT03839771). Ivosidenib was combined with azacytidine in another phase 3 trial (NCT03173248), resulting in a 53% CRc rate in patients with ND IDH1-mutant AML [105]. Common grade ≥ 3 AEs included febrile neutropenia and neutropenia [105].

IDH mutations have been also shown to induce BCL-2 dependence, increasing susceptibility to BCL-2 inhibition [52]. A clinical trial of ivosidenib combined with venetoclax (NCT03471260) showed an 83% CRc rate 83% with manageable grade ≥ 3 AEs, including febrile neutropenia and pneumonia, indicating this therapy is effective and well tolerated [52].

In the armamentarium of IDH1 inhibitors, recently approved olutasidenib showed promising efficacy in patients with R/R AML. A phase 1/2 trial (NCT02719574) of olutasidenib as monotherapy or combined with azacitidine yielded CRc rates of 32% and 15%, respectively [95]. The most common grade ≥ 3 AEs were thrombocytopenia and febrile neutropenia [95]. These results supported further evaluation.

Other IDH1 inhibitors, BAY1436032 and IDH305, demonstrated robust activity in preclinical trials and were evaluated in phase 1 studies (NCT03127735, NCT02381886) [99, 106]. Although both drugs demonstrated good safety profiles, the overall response rates were low and did not support further development [99, 106].

The IDH2 inhibitor enasidenib was approved following a phase 3 trial (NCT02577406) demonstrating a 29.7% CRc rate in patients with R/R IDH2-mutant AML [94]. Enasidenib was also combined in phase 1 clinical trial with standard chemotherapy (NCT02632708), and 74% patients with ND IDH2-mutant AML achieved CRc [104]. In a clinical trial of enasidenib combined with azacytidine (NCT03683433), the CRc rate was 58% in patients with R/R IDH2-mutant AML and 100% in those with ND IDH2-mutant AML [100]. The most common grade ≥ 3 AE in most studies of enasidenib was increased blood bilirubin [94, 100, 104].

Although IDH1 and IDH2 inhibitors proved efficacious, isoform switching between mutant IDH1 and mutant IDH2 occurs and is resistant to selective IDH1 and IDH2 inhibitors [96]. Vorasidenib (AG-881), a dual IDH1/2 inhibitor, prevents isoform switching; however, the study of vorasidenib in AML was discontinued due to its limited efficacy in patients with R/R AML in a phase 1 trial (NCT02492737) [107].

Other dual IDH1/2 inhibitors, HMPL-306 and LY3410738, demonstrated potent antileukemic effects in AML PDX models [108, 109], leading to ongoing phase 1 studies of these drugs in patients with R/R AML (NCT04764474, NCT04272957, NCT0460300). Initial results found no dose-limiting toxicities [108, 109]. HMPL-306 demonstrated promising efficacy, supporting further investigation, while LY3410738 showed promising results only in IDH-inhibitor naïve patients [108, 109].

Besides IDH inhibitors, another agent targeting the TCA cycle, lipodate analogue CPI-613 (devimistat), was investigated in AML. CPI-613 inhibits both pyruvate dehydrogenase (PDH) and α-ketoglutarate dehydrogenase. In a preclinical study, CPI-613 reduced mitochondrial respiration and induced ROS production and mitophagy in AML cells [110]. In a clinical trial (NCT01034475), CPI-613 monotherapy demonstrated acceptable safety but limited efficacy [110]. However, in a phase 1/2 trial (NCT02484391), the combination of CPI-613, cytarabine, and mitoxantrone in older patients with R/R AML achieved a 44% CRc rate with good tolerability [110, 111]. These results supported further evaluation in the phase 3 ARMADA 2000 study (NCT03504410), but it was recently closed for futility [110, 112].

Recently reported preclinical results showed that inhibition of another TCA cycle enzyme, the succinyl dehydrogenase (SDH, ETC complex II), using inhibitors such as thenoyltrifluoroacetone (TTFA) or 3-nitropropionic acid (3-NPA) combined with MCT1 lactate transporter inhibition by α-cyano-4-hydroxycinnamate (CHC) or AZD3965 impaired leukemogenesis in FLT3-ITD mutated AML models in vitro and in vivo [113], warranting further investigation.

### Amino Acid Metabolism

To meet the biosynthetic demands of the fast-growing leukemic cells, their metabolism is rewired toward acquisition of AAs from the tumor microenvironment (TME) [18, 19]. This leads to AA deficiency in the TME and decreases the proliferation of immune effector cells, promoting tumor growth [114].

Glutamine plays a special role for leukemic cells due to its ability to form glutathione, which enables scavenging ROS-induced damage, provides αKG entering the TCA cycle, and serves as the main energy source for AML blasts [15, 17, 19, 115-120].

Depleting glutamine in culture media decreases its catabolism, reduces leukemic cell proliferation, and induces apoptosis in AML cell bulk [15, 121]. This depletion can be achieved by asparaginases, enzymes that catalyze the hydrolysis of asparagine and exhibit glutaminase activity, leading to glutamine deprivation [15, 121-124]. While antitumor therapy with asparaginases is well established, its administration was associated with serious AEs, including hepatic disorders, coagulopathy, and pancreatitis [125]. To address these issues, new asparaginase formulations such as Pegcrisantaspase (PegC), a long-acting polyethylene glycol (PEG)-conjugated crisantaspase, were developed. Due to reports showing that interference with glutamine metabolism in AML overcomes resistance to BCL-2 inhibition, PegC combined with venetoclax was evaluated in a preclinical study [126]. Promising results led to the phase 1 trial of this combination in R/R AML patients (NCT04666649). Despite low reported CRc rates of 29% for PegC/venetoclax [127] and 20% for PegC combined with fludarabine and cytarabine in patients with R/R leukemias (NCT04526795) [128], PegC improved outcomes in some heavily pretreated R/R AML patients. Another novel formulation, red blood cell-loaded asparaginase (GRASPA), was tested in a phase 2 trial combined with LDAC (NCT01810705). GRASPA did not improve the CR ratio nor overall survival [124].

Efforts to impair glutamine metabolism in AML were also achieved by glutaminase inhibition with CB-839 (telaglenastat). In preclinical studies, CB-839 induced OXPHOS reduction, suppressed AML cell proliferation, triggered cell death [15, 16, 118], and showed synergy with venetoclax and gilteritinib and induced differentiation in IDH1- and IDH2-mutant AML cells in vitro [16, 17, 118, 120]. A phase 1 trial of the safety and efficacy of CB-839 as monotherapy in patients with AML (NCT02071927) has been completed, but the results are still pending. However, a phase 1b/2 trial of CB-839 combined with azacitidine in patients with myelodysplastic syndromes (NCT03047993) showed promising efficacy, with an ORR of nearly 70% [17].

Given the significantly higher expression of glutamine transporters LAT1, LAT3, and ASCT2 (SLC1A5) in AML cells versus normal cells, the antileukemic effect of inhibiting these transporters was tested [129-131]. LAT1/3 inhibitor 2-aminobicyclo-(2,2,1)-heptane-2-carboxylic acid (BCH) demonstrated promising antileukemic effect in vitro [18, 130, 132, 133]. Other LAT1 inhibitors, 2-amino-3(4[methoxy]-3,5-dichlorophenyl)propanoic acid (JPH203) and (S)-2-amino-3-(4-((7-(3-aminophenyl)naphthalen-1-yl)methoxy)-3,5-dichlorophenyl)propanoic acid (SKN103), also demonstrated antitumor effects, but their efficacy has not been tested in AML [129]. Pharmacological inhibition of SLC1A5, using GPNA or ASCT2 knockout, reduced leukemia progression in PDX models [134]. Notably, although ASCT2 expression is necessary for HSC function in stress hematopoiesis, it has limited impact on steady-state normal blood cells [134, 135]. A more potent ASCT2 inhibitor, V-9302, demonstrated a strong antitumor effect in mouse models of solid tumors and in AML cell lines [135, 136].

Glutamine metabolism can be also targeted using glutamine antagonists that inhibit variety of glutamine-metabolizing enzymes [137]. Acivicin and 6-diazo-5-oxo-norleucine (DON) alone each showed antileukemic effects, and DON treatment significantly improved AML cell eradication in mice harboring AML pretreated with induction chemotherapy [138]. Formulation of DON as a prodrug, administered in a low daily dosing regimen, demonstrated a potent antitumor effect without significant toxicity, presenting a rationale for further drug development and clinical testing [137].

Arginine is a multifunctional AA essential for synthesis of nitric oxide, urea, ornithine, and citrulline and plays a significant role in immunoregulation [139, 140]. Exogenous arginine is not necessary for normal cell growth but essential for leukemic cells, which often exhibit deficiency of argininosuccinate synthetase (ASS) [141]. The efficacy and safety of ADI-PEG20, which causes arginine depletion in patients with R/R AML, were evaluated in a phase 2 trial (NCT01910012). Preliminary results showed CR in 4.7% of all patients and 9.5% in patients with confirmed ASS-deficient AML [140]. Although the toxicity of ADI-PEG20 was minimal, ASS deficiency alone was insufficient to achieve responses in AML patients [140]. Therefore, a new trial assessing the efficacy of ADI-PEG20 combined with venetoclax and azacytidine (NCT05001828) is ongoing.

BCT-100 (pegylated arginase) also led to arginine depletion in the TME and showed high efficacy as monotherapy and combined with cytarabine in AML PDX models [142, 143]. A clinical trial assessed the antileukemic activity of BCT-100 combined with LDAC in older AML patients (ISRCTN40571019) but found no survival benefit [142, 143].

Another arginine-related target, protein arginine methyltransferase 5 (PRMT5), is involved in posttranslational modifications of arginine, and its downstream proteins showed oncogenic effect by regulating the expression of TP53, c-Myc, and FOXP3 [144]. The PRMT5 inhibitor LLY-283 combined with FLT3 TKIs showed synergistically enhanced inhibition of FLT3-ITD + AML cell lines compared to TKIs or PRMT5 inhibitor alone, warranting further investigation [145, 146].

Another essential AA, tryptophan, plays an important role in the immune response, and its level is tightly controlled by indoleamine 2,3-dioxygenase (IDO) [147, 148]. Overexpression of IDO was reported in AML blasts, leading to upregulation of tryptophan catabolism, and resulting in tryptophan depletion in the TME causing T-cell tolerance, immunosuppression, and promotion of tumor growth [149]. Indoximod, a small-molecule IDO inhibitor, was shown to impair IDO activity and thus reverse immunosuppression [149]. A phase 1 study (NCT02835729) of indoximod combined with standard induction chemotherapy in patients with ND AML demonstrated a 79% CRc rate, with febrile neutropenia and hypoxia being the most frequent grade ≥ 3 AEs [149]. Indoximod with standard AML induction therapy was well tolerated, supporting its further clinical evaluation [149].

Interestingly, FLT3-ITD occurs in 30% AML patients and was recently shown to promote serine synthesis [150]. Serine is a nonessential AA necessary for numerous metabolic pathways, including nucleotide synthesis [150]. A preclinical study reported that pharmacologic inhibition of serine biosynthesis by WQ-2101 selectively sensitized FLT3-ITD AML cells to cytotoxic chemotherapy in vitro and in vivo; however, the special role of serine in FLT3-mutant AML has not been clearly defined [150].

Cysteine is a nonessential AA imported by AML cells via the glutamate-cystine antiporter (xCT) to meet their biosynthetic demands in the form of glutathione synthesis [151]. Therefore, depletion of cysteine increases the ROS level and induces a non-apoptotic ROS-dependent cell death, ferroptosis [152, 153]. Sulfasalazine, a broadly available medicine with low toxicity profile, can inhibit xCT expression [152, 153] and demonstrated promising antileukemic effect when combined with standard chemotherapy, especially in NPM1-mutated AML samples [152, 153], leading to phase 1/2 clinical trial in elderly patients with ND AML (NCT05580861). Erastin, a more potent inhibitor of xCT, also induced ferroptosis and increased the sensitivity of AML cell lines to chemotherapeutic agents [154, 155]. However, the safety and efficacy of erastin in patients requires further investigation. Sorafenib, a kinase inhibitor approved for the treatment of hepatocellular carcinoma and advanced renal cell carcinoma, also inhibits xCT, and clinical trials have evaluated its efficacy in AML treatment (ACTRN12611001112954, NCT02474290). Although sorafenib reduced relapse rates in patients with FLT3-ITD AML, the mechanism of ferroptosis induction in AML cells remains unclear [156-158].

### Fatty Acid Metabolism

FAO is required for LSCs to remain dormant [14], and high expression of FAO genes is associated with increased resistance to venetoclax and cytarabine [14, 78, 159]. Pharmacological inhibition of CPT1 (Carnitine palmitoyltransferase I), a rate-limiting enzyme of FAO, induces apoptosis of AML cells through disrupting metabolic homeostasis and increasing ROS levels [160]. Although the CPT1 inhibitor etomoxir was broadly used in preclinical studies, its clinical development has been abrogated because of serious AEs [161]. Avocatin B, an avocado-derived lipid, is a more promising FAO inhibitor that demonstrated a good safety profile in a clinical study [162, 163]. Notably, co-culture with bone-marrow derived stromal cells reduced avocatin B’s antileukemic effect by increasing glucose and free fatty acid uptake [160, 162, 163], prompting studies of combination regimens with induction chemotherapy for AML, in which avocatin B enhanced sensitivity to chemotherapy targeting AML cells [160]. Another promising CPT1 inhibitor is ST1326, which induced apoptosis, cell growth arrest and mitochondrial damage in primary AML cells [164].

AYNE—a novel small-molecule inhibitor of very long-chain acyl-CoA dehydrogenase (VLCAD), which is essential both in FAO and OXPHOS—also reduced ATP production in AML cells, leading to a significant decrease of leukemia burden in preclinical models, sparing normal HSCs [165].

A recent preclinical study revealed that FAO inhibition can be also achieved by combining RNA-directed nucleoside analog 8-chloroadenosine (8-Cl-Ado) with venetoclax, which led to potent antileukemic activity [166]. For this reason, a phase 1 trial of venetoclax with 8-Cl-Ado has been initiated and it is currently recruiting (NCT05263284).

Interestingly, a recent study revealed that genetic or pharmacologic inhibition of fatty acid desaturases 1 and 2 (FADS1 and FADS2) synergized with venetoclax and azacitidine treatment and significantly reduced the viability of primary AML samples [14, 167]. This effect was derived because fatty acid desaturases function to increase NAD + recycling, leading to LSC survival during chemotherapy [167, 168].

Finally, other approaches addressing elevated expression of fatty acid receptors such as CD36, known to be associated with poor patient outcomes and chemoresistance, demonstrated promising results in preclinical studies [159, 169-171].

### Other Therapies Targeting Mitochondrial Metabolism

Although leukemic cells are dependent on OXPHOS, they also use glycolysis to maintain high metabolic demand, especially after OXPHOS inhibition [12, 172, 173]. Glycolytic cells prevent intracellular acidification by excreting excess lactate via the monocarboxylate transporters (MCTs) [173, 174]. MCT1 and MCT4 are overexpressed in leukemic cells, and MCT4 expression has been associated with poor prognosis in AML patients [173, 174]. Interestingly, pharmacological inhibition of MCT1 or MCT4 impaired the proliferation of leukemic cells in vitro [173, 174]. Such effect is associated with ability of leukemic cells to use lactate as an alternative carbon source to maintain mitochondrial respiration [173, 175]. These results reveal that inhibition of lactate utilization by AZD3965 or AZD0095 might be a promising strategy for AML treatment.

An old drug, arsenic trioxide (ATO), can affect mitochondrial metabolism, generate ROS, and downregulate BCL-2 expression [176]. ATO monotherapy showed good efficacy in acute promyelocytic leukemia (APL) but not in non-APL, even combined with chemotherapeutic drugs, however combined with venetoclax, demonstrated synergistic antileukemic effect in vitro, providing a rationale for further investigation [177]. Additionally, ATO combined with the selective FLT3 TKI sorafenib effectively eliminated FLT3/ITD + leukemic cells in vitro and in vivo, suggesting this combination as a potential candidate to study in clinical trials [178].

Heat shock protein-90 (Hsp90) is a chaperon molecule that stabilizes the mitochondrial proteome in neoplastic cells by reducing ROS production and thus preventing cell death [179]. Gamitrinib (GA mitochondrial matrix inhibitor) couples the Hsp90 inhibitor 17-allylamino-geldanamycin (17-AAG) to the mitochondrial-targeting moiety triphenylphosphonium (TPP). Gamitrinib demonstrated good antitumor efficacy and an acceptable safety profile in preclinical studies [179]. Gamitrinib also induced apoptotic cell death in AML blasts in vitro [180]. A clinical trial of gamitrinib in patients with lymphoma is ongoing (NCT04827810), but its efficacy in AML patients has not been established yet [179].

Dihydroorotate dehydrogenase (DHODH), an enzyme necessary in pyrimidine biosynthesis, was shown to be a metabolic regulator of cell differentiation, and its inhibition by brequinar induced differentiation of AML cells in vitro and in vivo, showing therapeutic potential [181, 182]. Novel DHODH inhibitors such as AG636, JNJ-74856665, PTC299, BAY2402234, and ASLAN003 also demonstrated antileukemic activity in preclinical models. Clinical trials with BAY2402234 (NCT03404726), ASLAN003 (NCT03451084), brequinar (NCT03760666) and JNJ-74856665 (NCT04609826 in patients with AML were terminated due to limited efficacy [183]. Interestingly, DHODH inhibitors led to degradation of FLT3-ITD protein and demonstrated a potent antileukemic activity in combination with quizartinib in quizartinib-resistant cells in preclinical models [184].

## Conclusions

Despite many new findings and better understanding of AML blasts biology in recent years, cytotoxic chemotherapy has remained the standard of care for AML. However, numerous studies highlighted the importance of differences in mitochondrial metabolism between normal and leukemic cells, indicating these alterations as a potential target in AML treatment. Targeting rewired metabolism could demonstrate antileukemic activity without affecting healthy cells. Thus, many promising compounds targeting these changes, such as venetoclax, MCL-1 inhibitors, IACS-010759, CPI-613, or CB-839 were developed. However, only few of these have proven safe and effective in AML treatment. Venetoclax has a good safety profile, but some AML cells acquire resistance to BCL-2 inhibition [8, 51, 52, 65, 69, 72]. Venetoclax-resistant cells can be eradicated by the addition of MCL-1 inhibitors [70]. Unfortunately, MCL-1 inhibition reduces the functionality of cardiomyocytes and suppress hematopoiesis [70]. IACS-010759, a potent OXPHOS inhibitor, showed dose-limiting toxicities in a clinical trial, which obstructed efforts to maintain effective antitumor activity [12]. CPI-613 showed a good safety profile in clinical trials, but its efficacy was limited [110, 112]. Glutaminase inhibitors such as CB-839 have limited activity when used as monotherapy, due to the development of compensatory mechanisms and/or resistance [16, 17, 118, 120]. Therefore, the most promising approach in AML therapy is the development of new drug combinations with synergistic activity [1]. AML-targeted combinations, especially those comprising drugs with different mechanisms of action, such as venetoclax, ivosidenib, and azacytidine, showed acceptable safety profiles and excellent response rates [52]. Alternative approach can be focused on inhibiting multiple metabolic pathways due to the AML blasts’ ability to restore reduced OXPHOS capacity using alternative energy sources such as fatty acids, glutamine, or lactic acid [14, 18, 113]. Finally, the promise of long-term remission after AML treatment will be fulfilled only when drug regimens effectively eradicate OXPHOS-dependent LSCs, which are a reservoir for relapse and resistance, and/or restore an immunosuppressive microenvironment of AML bone marrow. Therefore, compounds already tested safe in other indication like Tamoxifen in Breast Cancer with proven OXPHOS inhibitory activity tested in MPN, or new compounds like Ammocidin considered tolerable and effective in AML, could constitute a feasible alternative to mitochondrial complex I- or other TCA-inhibitors. Given however previously unknown toxicities occurred first in clinical trials such as lactate acidosis or neuropathy in IACS-010759 trial, more extensive preclinical studies are warranted and if proven non tolerable, indirect ways (like AA depletion, kinase inhibitors) might be considered as an achievable therapeutic strategy.

## Figures and Tables

**Table 1:** List of clinical trials showing clinical benefits of drugs and their combinations targeting mitochondrial metabolism in acute myeloid leukemia

Clinical trial	Target	Drugs	Indication	Phase	Status	Most common grade 3/4 AEs	CRc	Ref.
NCT03069352	BCL-2, DNA replication	VEN, AraC	AML unfit for IC	III	Active	Neutropenia (49%), thrombocytopenia (46%), febrile neutropenia (32%)	48%,	[67]
NCT02993523	BCL-2, DNA methylation	VEN, AZA	TX Naive AML unfit for IC	III	Active	Thrombocytopenia (46%), febrile neutropenia (43%), neutropenia (43%)	66%	[51]
NCT03661307	BCL-2, DNA methylation, FLT3	VEN, DEC, quizartinib	R/R or ND FLT3 mutated AML	I/II	Active	Pneumonia (73%), febrile neutropenia (53%), sepsis (18%) in R/R cohort pneumonia (40%), febrile neutropenia (50%), sepsis (10%) in frontline cohort	68%	[69]
NCT02115295	BCL-2, DNA replication, FLT3	VEN, AraC, cladribine, gilteritinib, IDA, midostaurin	ND AML	II	Active	Febrile neutropenia (84%), infection (12%), ALT elevations (12%)	94%	[5]
NCT03625505	BCL-2, FLT3	VEN, gilteritinib	R/R FLT3 mutated AML	Ib	Completed	Febrile neutropenia (44%), leukopenia (36%), platelet count decreased (25%), anemia (25%)	75%	[68]
NCT03471260	BCL-2, DNA methylation, IDH1	VEN, AZA, IVO VEN, IVO	ND or R/R AML IDH1 +	Ib/II	Active	Febrile neutropenia (28%), pneumonia (24%)	90% 80%	[52]
NCT02074839	IDH1	IVO	ND or R/R AML	I	Recruiting	Prolongation of the QT interval (7.8%), DS (3.9%), thrombocytopenia (3.4%)	30%	[103, 185]
NCT02632708	IDH1/2, DNA replication	IVO, ENA, AraC, daunorubicin, IDA, mitoxantrone, etoposide	ND AML IDH1 + or IDH2 +	I	Active	IVO - Hypophosphatemia (17%), hypokalemia (13%) and prolonged QT (10%)	IVO – 78%	[104]
						ENA - Blood bilirubin increased (16%), rash (14%), hypophosphatemia (13%)	ENA – 74%	
NCT03173248	IDH1, DNA methylation	IVO, AZA	ND AML IDH1+	III	Active	Febrile neutropenia (28%), neutropenia 27%)	53%	[105]
NCT02719574	IDH1	OLU OLU, AZA	R/R AML IDH1+	I/II	Completed	OLU - thrombocytopenia (28%), febrile neutropenia (22%), anemia (22%)	32%	[95]
						OLU + AZA - thrombocytopenia (41%) febrile neutropenia (28%), neutropenia (28%)	15%	
NCT02577406	IDH2	ENA	R/R AML IDH2+	III	Completed	Thrombocytopenia (10%), increased blood bilirubin (8%), neutropenia (6%)	30%	[94]
NCT03683433	IDH2, DNA methylation	ENA, AZA	R/R and ND AML IDH2+ unfit for IC	II	Active	ND AML – Increased blood bilirubin (29%), febrile neutropenia (14%)	ND	[100]
						R/R AML - Increased blood bilirubin (37%), febrile neutropenia (26%)	AML – 100% R/R AML – 58%	
NCT04526795	Asparagine, glutamine, DNA replication	Pegcrisantaspase, fludarabine, AraC	R/R AML	I	Active	Febrile neutropenia (67%), TLS (7%)	20%	[127]
NCT02835729	IDO, DNA replication	Indoximod, AraC, IDA	ND AML	I	Completed	Febrile neutropenia (60%), hypoxia (16%), pneumonia (12%)	79%	[186]

AE, adverse events; ALT, alanine-aminotransferase; AML, acute myeloid leukemia; AST, aspartate aminotransferase; AZA, azacitidine; BCL-2, B-cell lymphoma 2; CRc, composite complete remission; DS, differentiation syndrome; AraC, cytarabine; DEC, decitabine; ENA, enasidenib; IDA, idarubicin; OLU, olutasidenib; IC, intensive chemotherapy; IDH1, isocitrate dehydrogenase 1; IDH2, isocitrate dehydrogenase 2; IVO, ivosidenib; ND, newly diagnosed; R/R, relapsed/refractory; FN, febrile neutropenia; TLS, tumor lysis syndrome; VEN, venetoclax

## Data Availability

No datasets were generated or analysed during the current study.
